# Patientenzufriedenheit bei Patienten mit rheumatoider Arthritis: Effekte der Delegation an rheumatologische Fachassistenz

**DOI:** 10.1007/s00393-025-01747-4

**Published:** 2025-11-03

**Authors:** Birte Luise Hägermann, Juliana Rachel Hoeper, Sara Eileen Meyer, Patricia Steffens-Korbanka, Torsten Witte, Dirk Meyer-Olson, Kirsten Hoeper

**Affiliations:** 1 Klinik für Rheumatologie und Immunologie, Medizinische Hochschule Hannover, Hannover, Deutschland; 2 rheumapraxis an der hase, Osnabrück, Deutschland; 3 Knappschaft Kliniken Westfalen, Klinik für Rheumatologie und klinische Immunologie, Kamen, Deutschland

**Keywords:** Versorgungsqualität, Rheumatologische Fachassistenz, Teambasierte Versorgung, Delegation, Ambulante Versorgung, Quality of care, Rheumatological specialist assistants, Team-based care, Delegation, Outpatient care

## Abstract

**Hintergrund:**

Die Zufriedenheit von Patient:innen mit rheumatoider Arthritis (RA) beeinflusst Krankheitskontrolle, Therapietreue sowie das körperliche und psychische Wohlbefinden – entscheidende Faktoren für den Langzeiterfolg. Da eine leitliniengerechte, patientenzentrierte Versorgung angesichts limitierter ärztlicher Kapazitäten nicht flächendeckend umsetzbar ist, wird der Einsatz rheumatologischer Fachassistenz (RFA) als Ergänzung untersucht.

**Fragestellung:**

Wie ist der Einfluss teambasierter Versorgung auf die Patient:innenzufriedenheit bei seropositiver RA im Krankheitsschub.

**Material und Methoden:**

In einer multizentrischen, pragmatischen randomisierten kontrollierten Studie über 12 Monate wurden 224 Patient:innen mit seropositiver RA eingeschlossen. Nach Basiserhebung fanden 5 Folgevisiten statt. In der Interventionsgruppe (IG) wurden 3 Visiten durch eine RFA durchgeführt, in der Kontrollgruppe (KG) erfolgte die Betreuung konventionell. Nach 12 Monaten erfolgte eine Subgruppenbildung nach Krankheitsaktivität (DAS28 < 2,6 vs. ≥ 2,6). Primärer Fokus lag auf der Patient:innenzufriedenheit (ZAP-Fragebogen).

**Ergebnisse:**

Nach 12 Monaten berichtete die IG signifikant höhere Zufriedenheit in den Dimensionen Interaktion (*p* = 0,023), Information (*p* = 0,014), Kooperation (*p* = 0,021), Behandlungsqualität (*p* = 0,005) und Vertrauen (*p* = 0,028). Für Praxisorganisation (*p* = 0,515) und globale Zufriedenheit (*p* = 0,084) ergaben sich keine Unterschiede. In der Subgruppe ohne Remission zeigten sich vergleichbare Effekte, nicht jedoch bei Patient:innen in Remission.

**Diskussion:**

Die teambasierte Versorgung hatte positiven Einfluss auf multiple Dimensionen der Patient:innenzufriedenheit bei aktiver RA.

## Abstract (en)

**Background:**

The satisfaction of patients with rheumatoid arthritis (RA) influences disease control, treatment adherence and both physical and psychological well-being, which are key factors for long-term treatment success. However, due to limited physician resources, guideline-based patient-centered care cannot be universally implemented. As a response, the integration of trained rheumatological specialist assistants (RFA) into the care is evaluated as a complementary approach.

**Objective:**

This study aimed to assess the impact of team-based care on patient satisfaction among individuals with seropositive RA during flares.

**Material and methods:**

In this 12-month multicenter, pragmatic randomized controlled trial, 224 patients were enrolled. Following baseline assessment, five follow-up visits were scheduled. In the intervention group (IG), three of these visits were conducted by RFAs, while the control group (CG) received standard rheumatologist-led care. After 12 months participants were stratified by disease activity (DAS28 < 2.6 vs. ≥ 2.6). A secondary outcome was patient satisfaction, assessed using the ZAP questionnaire.

**Results:**

After 12 months the IG reported significantly higher satisfaction in the domains of interaction (*p* = 0.023), information (*p* = 0.014), cooperation (*p* = 0.021), quality of care (*p* = 0.005) and trust in the healthcare provider (*p* = 0.028). No significant differences were found for practice organization (*p* = 0.515) or overall satisfaction (*p* = 0.084). Similar patterns were observed in the subgroup with active disease but not in patients in remission.

**Conclusion:**

Team-based care showed a positive impact on multiple dimensions of patient satisfaction in active RA.

Die Zufriedenheit der Patient:innen stellt einen wichtigen Qualitätsindikator in der rheumatologischen Versorgung dar [16] und gewinnt an klinischer und gesundheitsökonomischer Bedeutung [8]. Sie umfasst Dimensionen wie Wahrnehmung der Behandlungseffektivität, Kommunikation sowie Partizipation [7, 14]. Insbesondere personelle Versorgungsengpässe erschweren jedoch eine patientenzentrierte Betreuung [4]. Die Delegation ärztlicher Leistungen an rheumatologische Fachassistenten (RFA) gilt als effektiv und sicher [11, 13], der Effekt auf die Patient:innenzufriedenheit ist in Deutschland bislang kaum untersucht.

## Hintergrund und Fragestellung

Die Patient:innenzufriedenheit beschreibt die subjektive Wahrnehmung und Bewertung des Versorgungsprozesses durch die Patient:innen. Sie stellt ein komplexes, multifaktorielles Konstrukt dar, das durch individuelle, psychosoziale sowie strukturelle Faktoren beeinflusst wird. Sie korreliert u. a. mit der Therapieadhärenz, der Krankheitsaktivität und dem Gesundheitsverhalten [6, 8, 17, 22, 24]. Die Qualität der Behandler:in-Patient:innen-Interaktion, insbesondere Vertrauen, erwies sich als zentraler Faktor der Zufriedenheit [17]. Eine gute Beziehung stärkt dabei das Vertrauen in die Versorgung [29]. Als weitere zentrale Aspekte der Betreuung gelten Empathie, fachliche Spezialisierung, Informationsvermittlung, Zeit und Kontinuität [1]. Besonders wichtig ist eine verständliche, strukturierte Informationsvermittlung zu Diagnose, Therapie und Prognose, die Gesundheitskompetenz und das Gefühl von Sicherheit und Kontrolle über die individuelle Krankheitssituation fördert [26]. In diesem Zusammenhang erweist sich auch Shared-Decision-Making als relevantes Konzept mit positiven Effekten auf Zufriedenheit und Therapieerfolg [19]. Die Patient:innenzufriedenheit korreliert jedoch nicht zwangsläufig mit objektivierbaren klinischen Verbesserungen, sondern wird stark durch Komorbidität und psychische Faktoren beeinflusst. Zudem zeigen Patient:innen mit diskrepanten Erwartungen an den Behandlungserfolg häufig Unzufriedenheit, selbst bei messbarem Therapieerfolg [25]. Dies verdeutlicht die Notwendigkeit, neben Patient-Reported Outcome Measures (PROMs) auch Patient-Reported Experience Measures (PREMs) in die Evaluation der Patient:innenzufriedenheit einzubeziehen. PROMs erfassen krankheitsbezogene Veränderungen wie die Symptomlast, während PREMs Versorgungsaspekte wie Kommunikation, emotionale Unterstützung und Koordination abbilden [5].

Angesichts wachsender Anforderungen an patientenzentrierte Versorgungskonzepte bei gleichzeitiger Unterversorgung gewinnen interprofessionelle Modelle, insbesondere teambasierte Ansätze, an Bedeutung [4]. Die vorliegende Studie untersucht die Effekte einer strukturierten, teambasierten Intervention auf die Zufriedenheit in der ambulanten rheumatologischen Versorgung bei Patient:innen mit seropositiver rheumatoider Arthritis (RA) im Krankheitsschub.

## Studiendesign und Untersuchungsmethoden

Die Datengrundlage bildete die multizentrische, randomisierte, kontrollierte ERFASS-Studie („Effektivität der RFA-Sprechstunde“), ein Vergleich der teambasierten Versorgung mit Regelversorgung bei RA-Patient:innen. Eingeschlossen wurden Erwachsene mit seropositiver RA (ICD-10: M05.8) bei Beginn oder Anpassung (Eskalation/Wechsel) einer Therapie. Ausschlusskriterien waren fehlende 12-monatige Verfügbarkeit, schwere Begleiterkrankungen, unzureichende Deutschkenntnisse oder fehlende Einwilligungsfähigkeit.

Im Beobachtungszeitraum von 52 Wochen fanden 6 Visiten statt (Baseline, Woche 6, 12, 24, 36 und 52). In der IG wurden die Visiten zu Woche 6, 12 und 36 von einer RFA durchgeführt und durch einen ärztlichen Kurzkontakt ergänzt. Primärer Endpunkt war die Veränderung des „Disease Activity Score“ (DAS28) über 52 Wochen zur Prüfung der Nichtunterlegenheit [11]. Zu den sekundären Endpunkten zählte u. a die Patient:innenzufriedenheit, erhoben mit dem validierten ZAP-Instrument [2], das von der KBV für strukturierte Befragungen im Qualitätsmanagement bereitgestellt wird [12]. Der Fragebogen umfasst 23 Items zu 4 Dimensionen: Interaktion (8 Items), Information (8 Items), Kooperation (3 Items) und Praxisorganisation (4 Items), sowie 3 globale Bewertungen zur Behandlungsqualität, zum Vertrauen in die behandelnde Person und zur allgemeinen Zufriedenheit. Die Antworten werden auf einer vierstufigen Likert-Skala erfasst. Die Rohwerte werden standardisiert auf 0–100 transformiert. Eine Aggregation zu einem Skalengesamtwert, sowie die Einbeziehung der Globalbewertungen erfolgen nicht [3]. Die Erhebung der Patient:innenzufriedenheit erfolgte zu Baseline, Monat 6 und 12. Für eine Subgruppenanalyse zur Krankheitsaktivität (DAS-28) wurde das Kollektiv nach 52 Wochen in Remission (DAS28 < 2,6; *n* = 102) und Nichtremission (DAS28 ≥ 2,6; *n* = 77) unterteilt. Nach Rücksprache mit den Autor:innen des Fragebogens wurde die originale Formulierung „Arzt/Ärztin“ durch „Behandler/Behandlerin“ ersetzt.

## Visiteninhalte und Gruppenstruktur

In der IG wurden die Visiten in Woche 6, 12 und 36 strukturiert von einer RFA durchgeführt. Inhalte waren eine standardisierte Anamnese (checklistenbasiert), die Erhebung des DAS-28, ein Komorbidität-Screening sowie Abfrage zur Medikamenteneinnahme und Nebenwirkungen. Ergänzend wurden psychosoziale Aspekte wie Lebenssituation, psychische Belastungen, Erwerbsfähigkeit und Rehabilitationsbedarf berücksichtigt. Im Anschluss erfolgte ein ärztlicher Kurzkontakt; bei Bedarf eine Schulung zur Therapieanwendung. Die aufgewendete Zeit für die Visiten wurde erfasst. Teilnehmende der KG erhielten die rheumatologische Regelversorgung mit ärztlich geführten Konsultationen alle 3 Monate sowie einen kurzen Zusatzkontakt zur Umsetzung des Treat-to-Target-Prinzips. In beiden Gruppen konnten bei klinischem Bedarf zusätzliche Termine vereinbart werden.

## Fallzahlkalkulation

Die Fallzahlkalkulation für die sekundären Endpunkte basierte auf dem Wilcoxon-Vorzeichen-Rang-Test und wurde mit der Software G*Power 3 durchgeführt. Unter Annahme einer Effektgröße von d = 0,4, einem Signifikanzniveau von α = 0,025, einer Power von 95 % sowie einer angenommenen Dropout-Rate von 10 % wurde eine Mindestanzahl von 74 Patient:innen als erforderlich berechnet.

## Statistische Analysen

Die statistische Auswertung erfolgte mit IBM SPSS Statistics V.25. Die Verteilung metrischer Variablen wurde mittels Shapiro-Wilk-Test überprüft. Die Daten der ZAP-Analyse zeigten keine Normalverteilung, sodass der Mann-Whitney-U-Test verwendet wurde. Zur Prüfung der Veränderung über die Zeit und der Nichtunterlegenheit wurde ein vordefinierter Grenzwert für die Effektgröße von d = 0,4 verwendet, entsprechend dem in der Literatur etablierten „Minimal Clinically Important Difference“ (MCID) für patientenberichtete Endpunkte [20]. Ein *p* < 0,05 wurde für alle Tests als statistisch signifikant definiert.

## Ethische und administrative Aspekte

Die Studie wurde im Rahmen des vom Innovationsfonds geförderten Projekts Rheuma-VOR (#01NVF16029) als eigenständiges Subprojekt konzipiert. Sie erfolgte nach positiver Votierung durch die Ethikkommission der Medizinischen Hochschule Hannover (# 3638-2017) sowie in Übereinstimmung mit nationalen gesetzlichen Vorgaben und der Deklaration von Helsinki. Die Rheuma-Liga Niedersachsen e. V. war an allen Studienphasen beteiligt. Alle Patient:innen gaben nach Aufklärung ihr schriftliches Einverständnis. Die Studie wurde im Deutschen Register Klinischer Studien registriert (#DRKS00013055).

## Ergebnisse

**Abb. 1:**
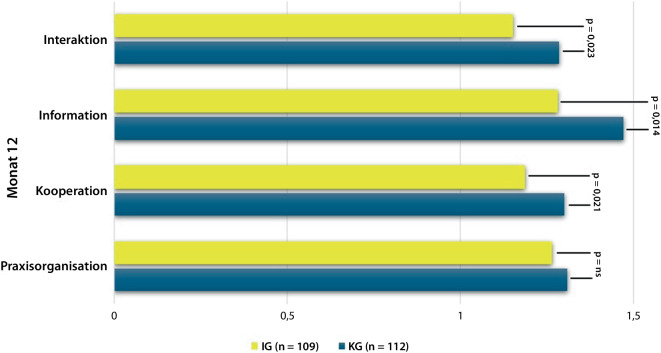
Patient:innenzufriedenheit der Dimensionen Interaktion, Information, Kooperation und Praxisorganisation zu Monat 12, Likert-Skala: 1 = sehr zufrieden bis 4 = sehr unzufrieden

Insgesamt wurden 224 Patient:innen randomisiert (KG *n* = 113; IG *n* = 111); die Drop-out-Rate betrug 8 % [11]. Die Baseline-Charakteristika sind in Tab. 1 dargestellt. Nach 12 Monaten zeigte die IG im Vergleich zur KG eine signifikant höhere Zufriedenheit in den Dimensionen Interaktion (*p* = 0,023), Information (*p* = 0,014) und Kooperation (*p* = 0,021), nicht jedoch in der Praxisorganisation (*p* = 0,515) (Abb. 1).

**Tab. 1:** Baseline Charakteristika der Studienpopulation stratifiziert nach Gruppe

	KG (*n* = 113)	IG (*n* = 111)	Gesamt (*n* = 224)
Weiblich, *n* (%)	86 (77)	80 (72)	166 (74)
Alter (Jahre), MW (SD)	58 (12)	59 (12)	59 (12)
RF-positiv (*n*)	105	101	206
ACPA-positiv (*n*)	96	96	192
Berufstätig, *n* (%) (112, 110)*	56 (49)	54 (49)	105 (47)
Schulbildung, n (%) (112, 110)*			
Keine weiterführende Schule	94 (83 %)	83 (75 %)	177 (79 %)
Weiterführende Schule	19 (17 %)	28 (25 %)	45 (21 %)
Ausbildung (111, 109)*, n (%)			
Keine	18 (16)	16 (15)	34 (16)
Berufsausbildung	85 (77)	79 (72)	164 (74)
Universitätsabschluss	14 (13)	14 (13)	22 (10)
*Therapieregime n (%)*			
Therapieeinstellung	41 (37)	28 (25)	69 (31)
Therapieumstellung	41 (37)	49 (44)	90 (40)
Therapieeskalation	31 (27)	34 (31)	65 (29)
Krankheitsdauer, Jahre, Median	6 (3–13)	8 (3–25)	6 (3–26)
Therapie zur Baseline, n (%) (103, 104)*			
Glukokortikoide	39 (38)	39 (38)	78 (38)
Methotrexat (103, 105)*	35 (34)	41 (39)	76 (37)
Leflunomid	14 (14)	11 (11)	25 (12)
Sulfasalazin	4 (4)	6 (6)	10 (5)
Hydroxychloroquin	2 (2)	2 (2)	4 (2)
JAK-Inhibitoren	1 (1)	3 (3)	4 (2)
Biologika	22 (21)	27 (26)	49 (22)
Ergebnisse, Median (IQR):			
DAS28-CRP (110, 111)*	4,4 (3,5–5,1)	4,5 (3,4–5,2)	4,4 (3,5–5,2)
Druckschmerzhafte Gelenke	6 (2–10)	6 (2–10)	6 (2–10)
Geschwollene Gelenke	3 (1–6)	3 (1–6)	3 (1–6)
Patienteneinschätzung Krankheitszustand	60 (44–75)	60 (40–79)	60 (42–75)
**ZAP Fragebogen (*n*** **=** 112, 109)*, **Median (IQR)**			**U/z/p-Wert**
Dimension Information	1,25 (1,0–1,75)	1,25 (1,0–1,5)	5603/−1,074/0,284
Dimension Interaktion	1,0 (1,0–1,3438)	1,0 (1,0–1,25)	5685,5/−1,036/0,301
Dimension Kooperation (111, 108)*	1,0 (1,0–1,6667)	1,0 (1,0–1,33)	5386,5/−1,479/0,139
Dimension Praxisorganisation (112, 110)*	1,25 (1,0–1,75)	1,25 (1,0–1,5)	5850/−0,667/0,506
**ZAP Globaldimensionen, Median (IQR)**			
Vertrauen (112, 110)*	4 (3–4)	4 (4–4)	5274,5/−2,321/0,02
Qualität (110, 109)*	2 (2–3)	3 (2–3)	5260/−1,814/0,74
Zufriedenheit (110, 109)*	2 (2–3)	3 (2–3)	4962/−2,557/0,01

*ACPA* Antikörper gegen citrullinierte Proteine, *DAS 28-CRP* Disease Activity Score in 28 Gelenken gemessen mit CRP, *IG* Interventionsgruppe, *KG* Kontrollgruppe, *Median (IQR)* Median (Interquartilsabstand), *MW* Mittelwert, *RF* Rheumafaktor, *SD* Standardabweichung, *U* Mann-Whitney (U)-Statistik, *z* z-Score, *ZAP* Fragebogen zur Zufriedenheit in der ambulanten Versorgung

*Alle Angaben basieren auf den Daten der randomisierten Studiengruppen, sofern keine abweichenden Stichprobengrößen in Klammern angegeben sind

**Abb. 2:**
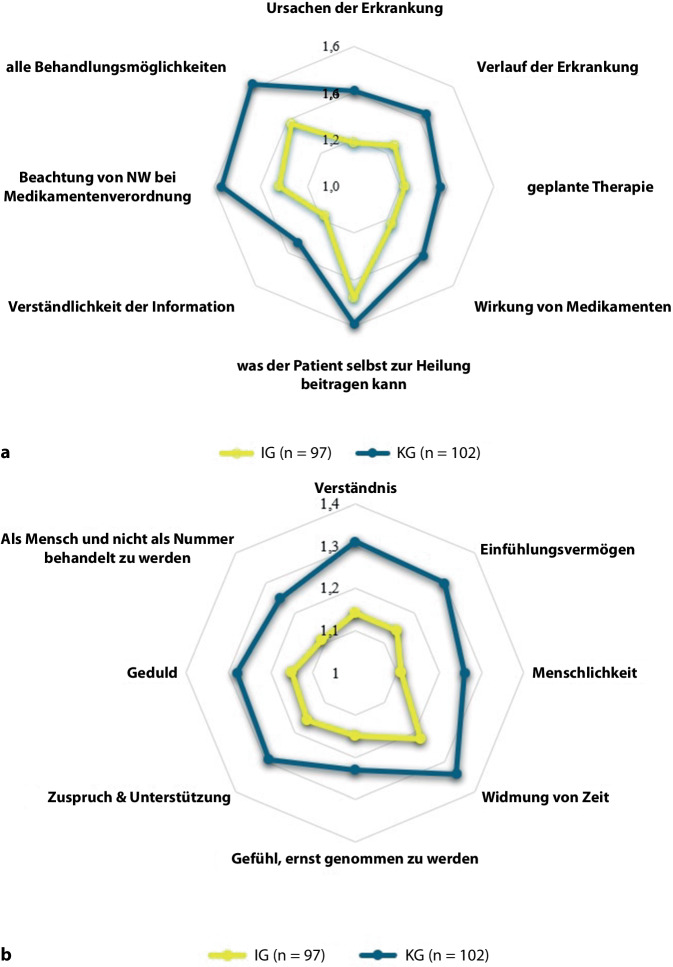
**a** Information: Einzelfragen zu Monat 12, Likert-Skala: 1 = sehr zufrieden bis 4 = sehr unzufrieden. **b** Interaktion: Einzelfragen zu Monat 12, Likert-Skala: 1 = sehr zufrieden bis 4 = sehr unzufrieden

Auf der Ebene einzelner Items der Dimension Information zeigten sich signifikante Unterschiede zugunsten der IG in 7 von 8 Bereichen: Ursachen (*p* = 0,001) und Verlauf (*p* = 0,012) der Erkrankung, Wirkung (*p* = 0,013) und Nebenwirkungen (*p* = 0,008) der Medikation, Informationen bezüglich der Berücksichtigung aller Behandlungsmöglichkeiten (*p* = 0,011), der Verständlichkeit der Informationen (*p* = 0,017) sowie der geplanten Therapie (*p* = 0,029). Lediglich das Item „was der Patient selbst zur Heilung beitragen kann“ zeigte keinen signifikanten Unterschied (*p* = 0,333) (Abb. 2a).

In der Dimension Behandler:in-Patient:innen-Interaktion bewertete die IG 6 von 8 Aspekten signifikant positiver: Verständnis (*p* = 0,008), Einfühlungsvermögen (*p* = 0,013), Menschlichkeit (*p* = 0,011) der behandelnden Fachperson, das Gefühl, „als Mensch und nicht als Nummer behandelt zu werden“ (*p* = 0,023), Zuspruch/Unterstützung (*p* = 0,037) sowie Geduld (*p* = 0,045) wurden in der IG besser bewertet. Kein signifikanter Unterschied zeigte sich beim Gefühl „ernst genommen zu werden“ (*p* = 0,181) und der gewidmeten Zeit (*p* = 0,087) (Abb. 2b).

Auch 2 von 3 Items der Kooperation wurden besser bewertet: Gründlichkeit und Sorgfalt der Untersuchungen (*p* = 0,033) sowie Zusammenarbeit mit anderen medizinischen Einrichtungen (*p* = 0,047). Kein Unterschied bestand bei der Überweisungsbereitschaft (*p* = 0,178). In der Dimension Praxisorganisation ergaben sich keine signifikanten Unterschiede (Wartezeit auf einen Termin: *p* = 0,401, Wartezeit in der Praxis: *p* = 0,535, Freundlichkeit des Personals: *p* = 0,917, Praxisatmosphäre: *p* = 0,544).

Bei der nach Krankheitsaktivität (Remission/Nicht-Remission) zu Monat 12 stratifizierten Subgruppenanalyse zeigten Patient:innen in Nicht-Remission (IG *n* = 35; KG *n* = 42) höhere Zufriedenheit in Interaktion (*p* = 0,047), Kooperation (*p* = 0,049), Behandlungsqualität (*p* = 0,044) und Vertrauen (*p* = 0,024), während für Patient:innen in Remission (IG *n* = 62; KG *n* = 60) keine Gruppenunterschiede bestanden (Tab. 2).

**Tab. 2:** ZAP: Dimensionen und Globaldimensionen, stratifiziert nach Krankheitsaktivität zu Monat 12

	KG Remission (*n* = 60) Median (IQR)	IG Remission (*n* = 62) Median (IQR)	Differenz			KG Nichtremission (*n* = 42) Median (IQR)	IG Nichtremission (*n* = 35) Median (IQR)	Differenz		
			U	z	*p*-Wert*			U	z	*p*-Wert*
**Dimensionen ZAP**										
Information	1,25 (1,0–1,75)	1,125 (1,0–1,375)	1540,5	−1,692	0,091	1,375 (1,0–2,0)	1,125 (1,0–1,5)	560,0	−1,833	0,067
Interaktion	1,0 (1,0–1,25)	1,0 (1,0–1,0)	1665,5	−1,261	0,209	1,0 (1,0–2,0)	1,0 (1,0–1,125)	563,5	−1,983	**0,047**
Kooperation	1,0 (1,0–1,33)	1,0 (1,0–1,0)	1658,0	−1,327	0,186	1,0 (1,0–2,0)	1,0 (1,0–1,125)	573,0	−1,983	**0,049**
Praxisorganisation	1,25 (1,0–1,5)	1,25 (1–1,5)	1823,5	−0,199	0,843	1,375 (1,0–1,5)	1,3 (1,0–1,5)	669,5	−0,700	0,490
**Globaldimensionen ZAP**										
Behandlungsqualität	3 (2–3)	3 (3–3)	1569,0	−1,937	0,067	3 (2–3)	3 (2–3)	569,5	−2,010	**0,044**
Vertrauen zum Behandelnden	4 (3–4)	4 (4–4)	1724,0	−0,956	0,392	4 (3–4)	4 (4–4)	570,0	−2,294	**0,024**
Allgemeine Zufriedenheit	3 (2–3)	3 (3–3)	1731,5	−0,881	0,403	3 (2–3)	3 (2–3)	607,5	−1,548	0,139

*IG* Interventionsgruppe, *KG* Kontrollgruppe, *Median (IQR)* Median (Interquartilsabstand), *U* Mann-Whitney(U)-Statistik, *z* z-Score, *ZAP* Fragebogen zur Zufriedenheit in der ambulanten Versorgung

*p-Werte: Signifikanzniveau *p* < 0,05, Unterschied zu Monat 12

**Abb. 3:**
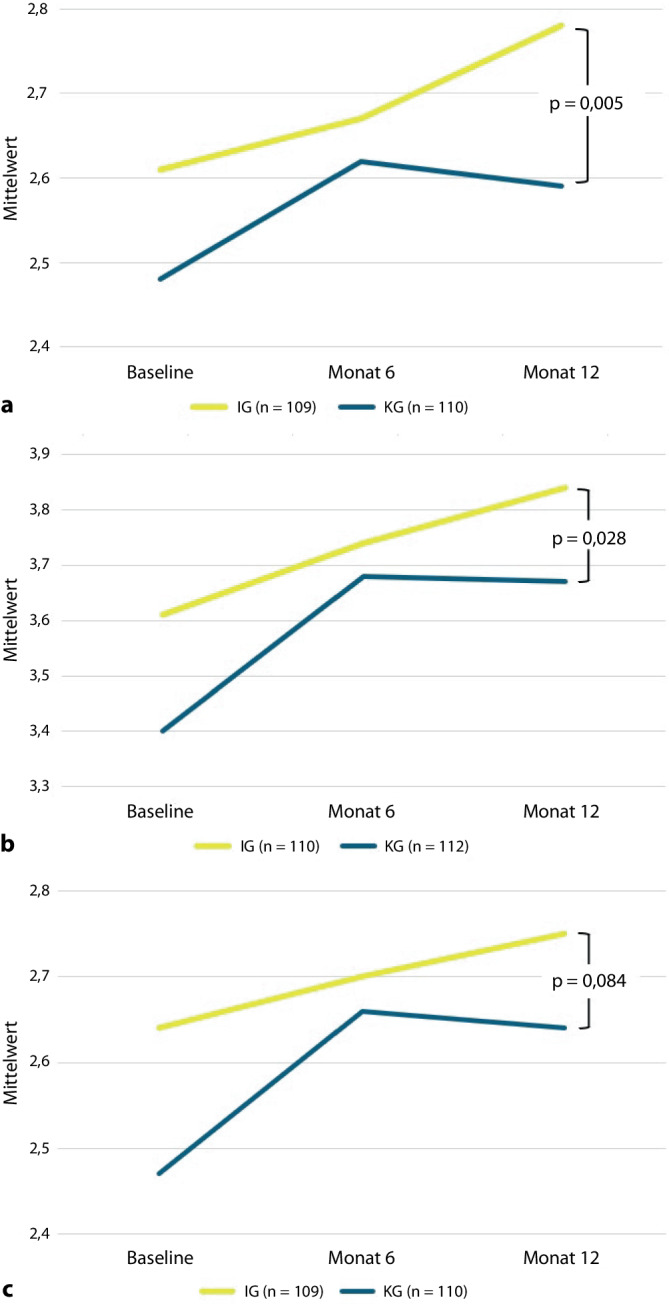
**a** Verlauf der Globaldimension: Qualität der Behandlung im Allgemeinen, Likert-Skala: 1 = sehr gering bis 4 = sehr hoch. **b** Verlauf der Globaldimension: Vertrauen in die behandelnde Person im Allgemeinen, Likert-Skala: 0 = Ich kenne den Behandler nicht lange genug, 1 = Nein, ich habe kein Vertrauen, 2 = Ich habe eher wenig Vertrauen, 3 = Ja, ich habe eher großes Vertrauen, 4 = Ja, ich habe großes Vertrauen. **c** Verlauf der Globaldimension: Zufriedenheit im Allgemeinen, Likert-Skala: 1 = sehr gering bis 4 = sehr hoch

Bei den 3 Globalfragen des ZAP (Qualität der Behandlung, Vertrauen, Zufriedenheit) zeigten sich keine signifikanten Unterschiede zwischen den Gruppen bei der Veränderung gemessen über 12 Monate [10]. Im direkten Vergleich zu Monat 12 schnitt die IG jedoch bei Behandlungsqualität (*p* = 0,005) und Vertrauen (*p* = 0,028), nicht aber bei allgemeiner Zufriedenheit (*p* = 0,084) besser ab (Abb. 3a–c).

## Diskussion

Die vorliegende Studie liefert Evidenz zum Einfluss einer strukturierten, teambasierten Versorgung mit Einbindung von RFA auf die Zufriedenheit von Patient:innen mit seropositiver RA im Schub. Die Ergebnisse zeigen eine signifikant höhere Zufriedenheit in mehreren Dimensionen zugunsten der IG (Abb. 1), insbesondere bei fehlender Remission nach 12 Monaten. Damit wird Patient:innenzufriedenheit als ergänzender Qualitätsindikator neben klinischen Endpunkten hervorgehoben.

In der Dimension Information war die Zufriedenheit in der IG signifikant höher, insbesondere bei krankheitsbezogenen Inhalten wie Ursachen, Verlauf sowie Wirkung und Nebenwirkungen der Therapie. Dies verdeutlicht den Mehrwert strukturierter, mehrzeitiger und repetitiver Aufklärung im interdisziplinären Team mit RFA. Auch Verständlichkeit und Darstellung aller Therapieoptionen wurden positiver bewertet, was auf ergänzende kommunikative Kompetenzen von RFAs und deren Beitrag zur Stärkung der Gesundheitskompetenz hinweist (Abb. 2a). Empathie, aktives Zuhören und Kommunikationsfähigkeit fördern die Patient:innenzufriedenheit und ein subjektives Sicherheitsgefühl [9, 21]. RFAs werden aufgrund ihres ganzheitlichen Ansatzes oft als vertrauensvolle Ansprechpartner:innen auf Augenhöhe wahrgenommen [15, 27]. Eine europäische Erhebung von Meisters et al. 2020 zeigte bestehende Versorgungslücken aus Patient:innensicht auf: unzureichende Information über Erkrankung und Therapieoption sowie fehlende Berücksichtigung individueller Bedürfnisse [18]. Die Ergebnisse der vorliegenden Studie verdeutlichen, dass teambasierte, interdisziplinäre Versorgung zur Schließung dieser Lücken beitragen kann.

Auch in der Dimension Behandler:in-Patient:innen-Interaktion war die Zufriedenheit in der IG signifikant höher, insbesondere bei zentralen Faktoren einer stabilen Behandlungsbeziehung wie Einfühlungsvermögen, Menschlichkeit und individueller Wahrnehmung. Kein Unterschied zeigte sich jedoch bei den Items „ernst genommen werden“ und „gewidmeter Zeit“ (Abb. 2b). Ausgehend von der Annahme, dass die Zufriedenheit der Patient:innen in der Regel mit der aufgewendeten Zeit für die individuelle Patient:innenbetreuung korreliert, ist dieses Ergebnis besonders hervorzuheben. Das könnte darauf hindeuten, dass die höhere Zufriedenheit nicht primär auf eine längere Gesprächsdauer von 20–30 min in der RFA-Visite zurückzuführen ist und die Patient:innen sich unabhängig vom Versorgungsmodell gleichermaßen respektiert fühlten. Dieses Ergebnis zeigt umso mehr das Potenzial einer Teamversorgung und einer sinnvollen Reallokation der Aufgaben, wie z. B. das Screening auf Komorbidität, was aktuell im Praxisalltag der Regelversorgung kaum umzusetzen ist. Mit durchschnittlich 7 min ärztlicher Kontaktzeit pro RFA-Visite [11] wird zugleich das Potenzial der RFA-Einbindung für eine ressourcenschonende, patientenzentrierte Versorgung deutlich.

In der Dimension Kooperation bewertete die IG die Gründlichkeit der Untersuchungen sowie die interdisziplinäre Zusammenarbeit signifikant besser, was sich durch die strukturierte Prozessführung und sorgfältige Dokumentation der RFA erklären könnte. Möglicherweise spiegelt die bessere Bewertung eher einen indirekten Effekt der intensiveren Betreuung wider als konkrete Maßnahmen.

In der Dimension Praxisorganisation zeigten sich keine signifikanten Unterschiede. Dies erscheint plausibel, da Terminvergabe, Wartezeiten und Praxisatmosphäre nicht durch die Intervention, sondern primär durch organisatorische Rahmenbedingungen beeinflusst werden.

Die Itemebene verdeutlicht die Breite der Interventionseffekte: In 15 von 23 Items erzielte die IG signifikant höhere Zufriedenheitswerte, besonders in der Dimension „Information“. Lediglich das Item „Was man selbst zur Heilung beitragen kann“ zeigte keinen Unterschied, was auf ungenutztes Potenzial zur Stärkung von Selbstwirksamkeit und aktiver Patientenrolle hinweisen könnte. Der Bedarf an individualisierter Beratung zu Lebensstil, Bewegung, Ernährung und psychosozialer Unterstützung wird offenbar unterschätzt und in standardisierten Konzepten unzureichend berücksichtigt [23].

Die Differenzierung nach Krankheitsaktivität zeigte, dass Patient:innen in Remission keine Unterschiede in den Gesamtdimensionen aufwiesen, vermutlich aufgrund insgesamt hoher Zufriedenheit und begrenzterem Zusatznutzen durch RFA. Patient:innen ohne Remission profitierten hingegen signifikant in den Dimensionen Interaktion, Kooperation sowie den Globalbewertungen „Vertrauen“ und „Behandlungsqualität“ (Tab. 2). Dies ist bemerkenswert, da hohe Krankheitsaktivität üblicherweise mit geringerer Zufriedenheit einhergeht [8, 28]. International wurde höhere Zufriedenheit vor allem bei Remission oder niedriger Aktivität beschrieben; allerdings schloss nur eine Studie Patient:innen mit hoher Krankheitsaktivität ein [6]. Angesichts des kleinen Subkollektivs (IG *n* = 35; KG *n* = 42) sind weitere Untersuchungen zur Belastbarkeit der Ergebnisse nötig. Dennoch deuten unsere Ergebnisse darauf hin, dass insbesondere Patient:innen mit anhaltend hoher Krankheitsaktivität von einem erweiterten Versorgungsansatz profitieren könnten.

Obwohl bei den Globalfragen keine Veränderung über die Zeit beobachtet wurde [10], zeigten sich zu Monat 12 signifikant höhere Werte für Vertrauen und wahrgenommene Behandlungsqualität in der IG. Dies stützt die Annahme, dass RFA als stabilisierender Faktor im Behandlungsteam wahrgenommen werden. Der Unterschied entwickelte sich vor allem zwischen Monat 6 und 12 (Abb. 3): Während Zufriedenheit mit Behandlungsqualität und Vertrauen in der IG weiter zunahmen, stagnierten oder sanken diese in der KG leicht. Eine anfängliche Zurückhaltung gegenüber der neuen Versorgungsform könnte den verzögerten Vertrauensaufbau erklären.

Die Studie weist Limitationen auf. Die Ergebnisse beziehen sich auf ein spezifisches Kollektiv mit seropositiver RA im Schub, wodurch die Übertragbarkeit begrenzt ist. Die Rekrutierung erfolgte zentrumsbasiert, wodurch ein Halo-Effekt nicht ausgeschlossen werden kann. Die Patient:innenzufriedenheit wurde ausschließlich mit dem selbstberichteten ZAP-Fragebogen erhoben, der anfällig für soziale Erwünschtheit, Recall-Bias und Kontexteinflüsse sein kann. Die 12-monatige Nachbeobachtungszeit erlaubt keine Aussagen zur Stabilität oder möglichen Veränderung der Effekte. Eine Langzeitbeobachtung wäre insbesondere für patientenzentrierte Outcomes wünschenswert. Die Änderung im ZAP-Fragebogen („Arzt/Ärztin“ → „Behandler:in“) wurde zwar autorenseitig freigegeben, stellt aber eine Abweichung vom Originalinstrument dar; der Einfluss auf das Antwortverhalten bleibt unklar.

Im internationalen Vergleich steht Deutschland bei der Einbindung von RFA in der Rheumatologie noch am Anfang. Die vorliegenden Ergebnisse sowie bisherige deutsche Studien zeigen jedoch, dass teambasierte Versorgung keinen Nachteil darstellt, sondern medizinisch wie patientenbezogen einen Mehrwert bietet [10, 11, 13]. Der Zugewinn an Zufriedenheit unterstreicht das Potenzial dieses Modells für eine qualitätsgesicherte Versorgung. Die nachhaltige Umsetzung dieses Modells ist jedoch durch finanzielle und strukturelle Faktoren limitiert. Die Qualifizierung von RFA erfordert zusätzliche Investitionen durch die entsprechende Weiterbildung. Darüber hinaus werden die zusätzlich erbrachten Leistungen im Rahmen der RFA-Visite bislang weder im Einheitlichen Bewertungsmaßstab noch im Gebührenverzeichnis für Privatversicherte vergütet. Die Kosten müssen daher von den Praxen selbst getragen werden. Hinzu kommt, dass höher qualifizierte RFA aufgrund ihres erweiterten Kompetenzprofils verständlicherweise eine höhere Vergütung erwarten, was die wirtschaftliche Belastung weiter verstärkt. Zudem ist qualifiziertes Fachpersonal nur begrenzt verfügbar. Eine langfristige Bindung der RFA ist angesichts des Fachkräftemangels im nichtärztlichen Bereich ebenfalls herausfordernd und eine kurzfristige Verbesserung der Personalsituation ist nicht absehbar. Diese Rahmenbedingungen erschweren derzeit die flächendeckende Implementierung einer teambasierten Versorgung, obwohl unsere Ergebnisse einen klaren Nutzen für Patient:innen zeigen.

## Fazit für die Praxis

- Teambasierte Versorgung mit Einbindung von rheumatologischer Fachassistenz (RFA) steigert die Patient:innenzufriedenheit.
- Die höhere Zufriedenheit betrifft v. a. die Informationsvermittlung sowie Aspekte der Interaktion.
- Die Vertrauensbildung ist nicht zwangsläufig auf längere Gesprächsdauer zurückzuführen, sondern auf qualitative Aspekte der Betreuung.
- Die Einbindung von RFA kann als strukturelle Ergänzung der rheumatologischen Regelversorgung empfohlen werden.
- Für eine flächendeckende Implementierung sind jedoch zukünftige Anpassungen, insbesondere der finanziellen Rahmenbedingungen dringend erforderlich.

## Data Availability

Die während dieser Studie generierten und analysierten Datensätze sind nicht öffentlich zugänglich. Die Daten sind jedoch von der korrespondierenden Autorin auf begründete Anfrage erhältlich.
